# Supplementary material for: Tunneling Spectroscopy for Electronic Bands in Multi-Walled Carbon Nanotubes with Van Der Waals Gap
Source: Molecules. 2021 Apr 7;26(8):2128. doi: 10.3390/molecules26082128 (PMC8068046; doi:10.3390/molecules26082128)
Supplement: Supplementary file 1 [file molecules-26-02128-s001.pdf]

molecules

Tunneling spectroscopy for electronic bands in multi-wall carbon nanotubes with van-der-Waals gap

Dong-Hwan Choi, Seung Mi Lee, Du-Won Jeong, Jeong-O Lee, Dong-Han Ha, Myung-Ho Bae and Ju-Jin Kim

Section 1. *sm*-MWCNT
1. AFM characterization

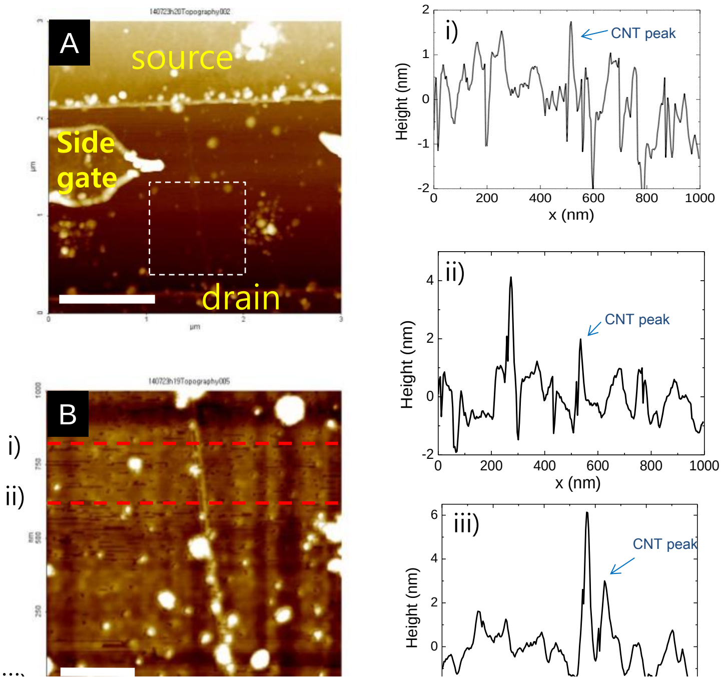

Figure S1. (a) AFM images of *sm*-MWCNT device. Scale bar: 2 μm. (b) AFM images of the dashed-box region in (a). Scale bar: 0.25 μm. The device has a side gate as well as the back gate. Right panel shows the height profiles at positions indicated by red dashed lines with i), ii) and iii) in (b), which shows that an average height (or diameter, *D*) of the MWCNT is ~ 3 nm.

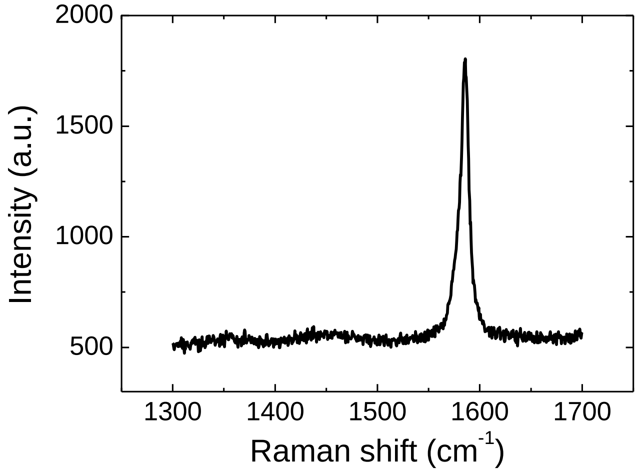

Figure S2. Raman shift of *sm*-MWCNT with a laser wavelength of 542 nm, which shows a single G peak at 1585.5 cm^{-1}. Usually, a single-wall CNT shows two G peaks with an interval of ~20 cm^{-1}, known as G^- and G^+ peaks [1]. Thus, the number of tube shell, n_s is at least > 1.

3. Band model for *sm*-MWCNT tunnel junction

Figure S3a shows a dI/dV_{sd} map as a function of V_{sd} and V_{bg} of *sm*-MWCNT at T = 4.2 K, which are the same with Figure 5b in the main panel. Figure S3b shows dI/dV_{sd}-V_{sd} curves for various V_{bg}. Figure S3c shows the comparison of dI/dV_{sd}-V_{sd} curve at V_{bg} = 0V (blue curve) with that obtained at V_{bg} = −40V (red curve), which is shifted in *x*-axis to match the minimum conductance to that of the blue curve as indicated by a blue arrow. As shown by several vertical dashed lines, the locations of the main conductance peaks in the two curves are consistent. Since a quantitative analysis based on calculations is difficult without detail information about the number of CNT shell and the kind of metal or semiconductor of inner CNT parts, we provide an intuitive way based on simple band structures as shown in Figure S3d and S3e. We start from an *e*-doped state with an energy gap (E_g), *e.g.*, $V_{bg} = V_{sd}$ = 0 V, as shown in the most left panel in Figure S3d, where the Fermi levels of source (E_{FS}) and drain (E_{FD}) are located at the same level between SC1 and SC2 subbands. We consider that the CNT is in a heavily *e*-doped state and the work function of CNT is larger than that of metal, resulting in a band diagram of the most left panel of Figure S3d. In that case, for slightly increasing V_{sd} in negative (middle panel of Figure S3d) and positive (the most right panel of Figure S3d) directions, E_{FS} meets the SC2 and SC1 subbands, respectively, resulting in conductance peaks as shown in Figure S43aan S3b for V_{bg} > 0 V. For further increasing V_{sd}, E_{FS} successively crosses other subbands, resulting in multiple conductance peaks. With negatively increasing V_{bg} for V_{bg} < 0 V, the bands in the CNT are started to be bended to an upper direction, and for certain V_{bg} conditions the Fermi levels of source and drain are located in the band gap of the CNT as shown in Figure S3e. At that condition, with positively increasing V_{sd}, E_{FS} meets the $E_g/2$ at first, resulting in the minimum conductance dip near zero-bias voltage as shown in Figure S3a and S3b for V_{bg} < −25 V with multiple conductance-peak structure. The energy gap, E_g estimated by the interval between SC1 and SV2 is ~0.13 eV, which is smaller than the expected E_g ~ 0.3 eV based on a relation of $\boldsymbol{E_g \approx 0.9\ \mathrm{nm} \cdot \mathrm{eV}/D(\mathrm{nm})}$ for a single-wall CNT case. The Raman spectrum suggests that the number of the shell of the CNT (n_s) is at least more than 1 (see Figure S2). We note that a CNT with n_s > 1 shows a hybridized E_g depending on electrical properties of inner shells. If a double-wall CNT (DWCNT) is considered for the simplicity, although the outer shell is the *sm*-CNT, E_g is even reduced as 2 factors if the

inner shell is a metallic (*m*-) CNT as shown in Figure 4b in the main text.

Figure S3. Band model to explain the observed multiple conductance peaks in the *sm*-MWCNT. Details are described in the text.

Section 2. *m*-MWCNT

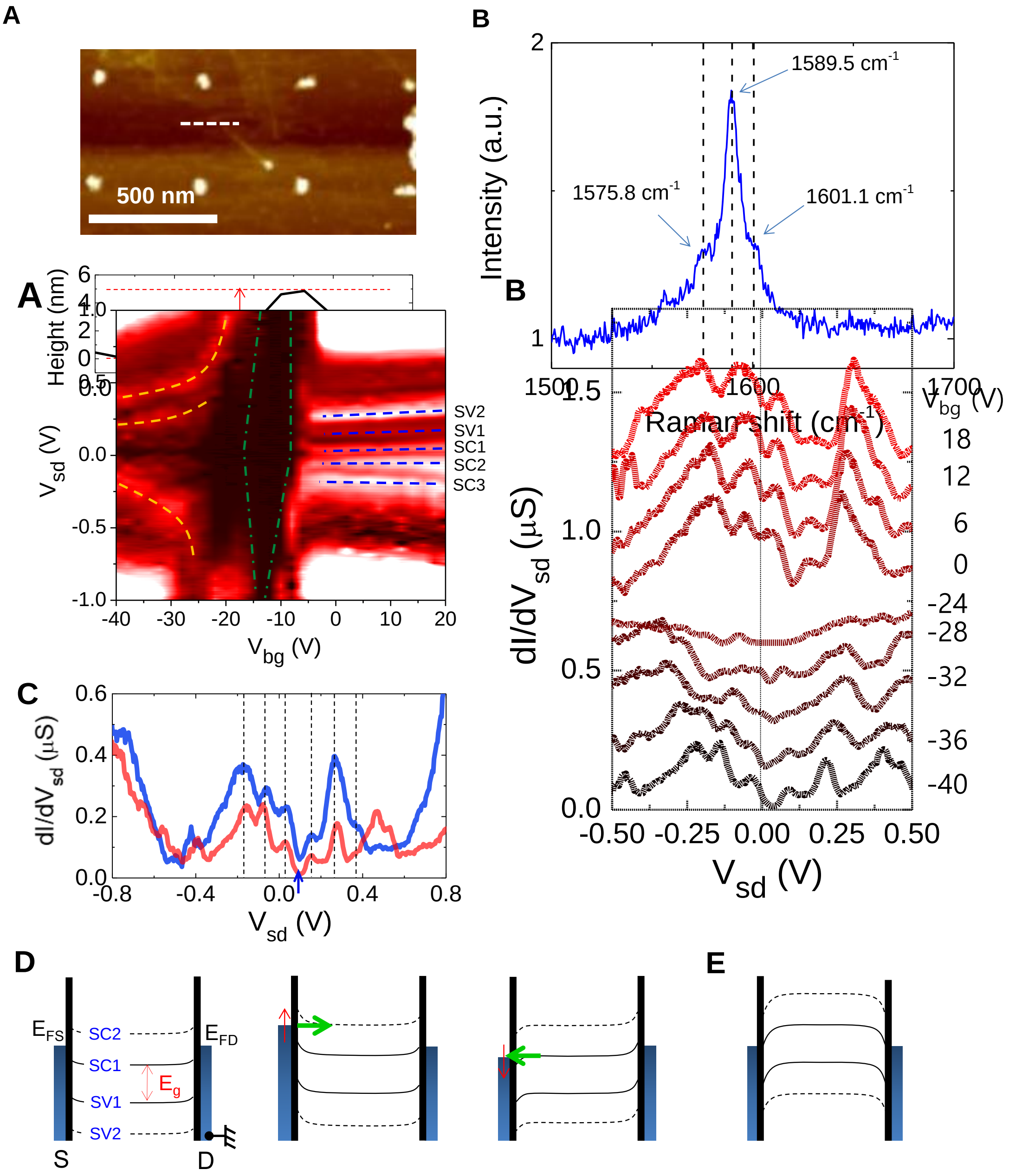

Figure S4. (a) AFM image of the *m*-MWCNT examined in Figure 6 in the main text, where the diameter of the CNT was estimated as ~4.8 nm. (b) Raman spectrum shows the main G peak at 1589.5 cm^{-1} with satellite peaks at 1575.8 and 1601.1 cm^{-1}, which are different from the characteristic of SWNT [1] and DWCNT (see Figure 1c in the main text).

References for supplementary materials

1. Kim, S. *et al.*, Oxygen plasma effects on the electrical conductance of single-walled carbon nanotube bundles. *J. Phys. D. Appl. Phys.* **2018,** 43, 305402.
